# Enhanced Nitrogen Loss by Eddy-Induced Vertical Transport in the Offshore Peruvian Oxygen Minimum Zone

**DOI:** 10.1371/journal.pone.0170059

**Published:** 2017-01-25

**Authors:** Cameron M. Callbeck, Gaute Lavik, Lothar Stramma, Marcel M. M. Kuypers, Laura A. Bristow

**Affiliations:** 1 Department of Biogeochemistry, Max Planck Institute for Marine Microbiology, Bremen, Bremen, Germany; 2 Department of Physical Oceanography, GEOMAR Helmholtz Centre for Ocean Research Kiel, Schleswig-Holstein, Germany; CAS, CHINA

## Abstract

The eastern tropical South Pacific (ETSP) upwelling region is one of the ocean’s largest sinks of fixed nitrogen, which is lost as N_2_ via the anaerobic processes of anammox and denitrification. One-third of nitrogen loss occurs in productive shelf waters stimulated by organic matter export as a result of eastern boundary upwelling. Offshore, nitrogen loss rates are lower, but due to its sheer size this area accounts for ~70% of ETSP nitrogen loss. How nitrogen loss and primary production are regulated in the offshore ETSP region where coastal upwelling is less influential remains unclear. Mesoscale eddies, ubiquitous in the ETSP region, have been suggested to enhance vertical nutrient transport and thereby regulate primary productivity and hence organic matter export. Here, we investigated the impact of mesoscale eddies on anammox and denitrification activity using ^15^N-labelled *in situ* incubation experiments. Anammox was shown to be the dominant nitrogen loss process, but varied across the eddy, whereas denitrification was below detection at all stations. Anammox rates at the eddy periphery were greater than at the center. Similarly, depth-integrated chlorophyll paralleled anammox activity, increasing at the periphery relative to the eddy center; suggestive of enhanced organic matter export along the periphery supporting nitrogen loss. This can be attributed to enhanced vertical nutrient transport caused by an eddy-driven submesoscale mechanism operating at the eddy periphery. In the ETSP region, the widespread distribution of eddies and the large heterogeneity observed in anammox rates from a compilation of stations suggests that eddy-driven vertical nutrient transport may regulate offshore primary production and thereby nitrogen loss.

## Introduction

Oceanic oxygen minimum zones (OMZ) typically occur in regions where upwelling of nutrient rich waters fuels high surface primary productivity. The resulting export of organic matter stimulates microbial respiration, and combined with poor regional ventilation creates low oxygen concentrations [1]. Traditionally OMZ boundaries are defined by oxygen concentrations of less than 20 μM [2], although, oxygen is regularly observed to be < 10 nM in these regions [3, 4]. Under low oxygen concentrations the anaerobic processes anammox and denitrification contribute to nitrogen loss. Specifically, the former catalyzes the anaerobic oxidation of ammonium with nitrite, while the latter is the stepwise reduction of nitrate to N_2_. An estimated 30–50% of oceanic nitrogen loss occurs in OMZs, which represent roughly 0.1% of the global ocean volume [5]. These regions are primarily located within the Arabian Sea, the Bay of Bengal, off the coast of Namibia, the Eastern Tropical North Pacific, and the Eastern Tropical South Pacific (ETSP) [6]. In the majority of OMZ studies, anammox has been shown to be the main sink of fixed inorganic nitrogen (NO_3_^-^, NO_2_^-^ and NH_4_^+^) [7–12]. The main source of inorganic nitrogen substrates for anammox comes from the remineralization of organic matter exported from the photic zone [13]. Based on *in situ* rate measurements, anammox activity is strongest over the upper shelf where the input of organic matter is highest [8, 9, 13]. Therefore, organic matter supply places constraints on nitrogen loss [13], which has been attributed to coastal upwelling [1].

The offshore OMZ (defined as >600m water depth following Kalvelage et al., [13]), despite having lower volumetric anammox rates (by an order of magnitude), accounts for two-thirds of ETSP nitrogen loss [13]. These rates are heterogeneous and not evenly distributed across the offshore ETSP region [13]. Consequently, there must be other mechanisms regulating nitrogen loss and potentially export production in the offshore OMZ [14], where coastal Ekman driven upwelling and the breaking of internal waves is less influential [15]. The most compelling suggestion is mesoscale eddies, which occur at a large-scale (50–200 km diameter), can persist for relatively long time periods (weeks to months), and are ubiquitous in the marine environment [16–18].

**Fig 1:**
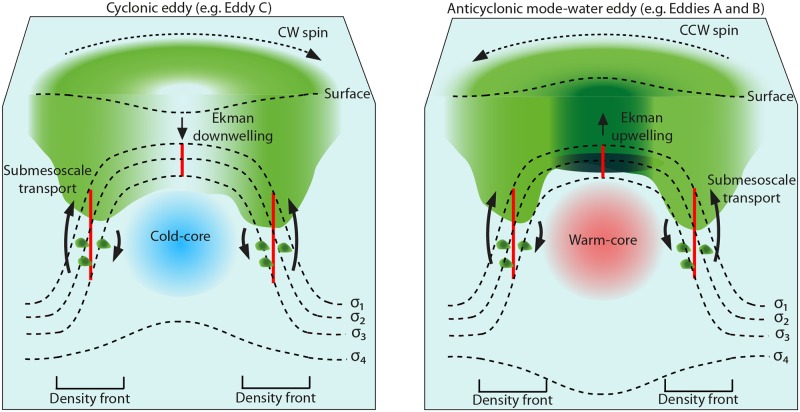
Schematic of isopycnal deformations and vertical transport processes in two eddy types depicted for the southern hemisphere. Shown are a cyclonic (clockwise ‘CW’ spin) and an anticyclonic mode-water eddy (counterclockwise ‘CCW’ spin). Both seasonal (dotted lines; σ_1–3_) and main pycnoclines (dotted line; σ_4_) are illustrated. At the surface, cyclonic and anticyclonic mode-water eddies cause a negative and positive sea surface height anomaly, respectively. Vertical transport processes in the eddy center can vary in direction and magnitude [20], here, we illustrate the direction of vertical transport in the eddy center derived from eddy-wind interaction Ekman flow. In the center of anticyclonic mode-water eddies, upwelling stimulates chlorophyll accumulation (green), in addition, inward swirling currents concentrate chlorophyll in the eddy center from surrounding waters, also known as eddy entrapment [20]. In contrast, cyclonic eddies distribute chlorophyll downwards through the eddy center. In both eddy types submesoscale vertical transport is expected to be enhanced along either side of the density front (i.e. along the tilted isopycnals). This area coincides with an increase in isopycnal spacing (red line) and the eddy horizontal velocity, which can be used to differentiate the eddy periphery from the eddy center. Submesoscale processes drive two-way vertical transport. A net upward transport of nutrients into the euphotic zone stimulates chlorophyll production, while subduction can act to re-distribute chlorophyll over a greater depth, and can cause chlorophyll pockets to form below the surface mixed layer. Submesoscale vertical velocities at the eddy periphery exceed velocities at the center, as represented by the length and thickness of the vertical arrows. This figure represents a synthesis of principles discussed by Mahadevan et al., [40]; Omand et al., [32]; and McGillicuddy [20].

Eddies mediate vertical advective transport of nutrients by Ekman and nonlinear Ekman mechanisms. Ekman transport is primarily driven by the eddy-wind interaction and is strongest in the eddy center [19, 20]. There are three main types of mesoscale eddies which can be characterized by their isopycnal displacements and the direction of Ekman-driven transport in the eddy center (Fig 1). In cyclonic eddies Ekman transport produces downwelling. In anticyclonic and anticyclonic mode-water eddies this generates upwelling [20–22], these two eddy types are distinguished by their differences in isopycnal displacements. In contrast, nonlinear Ekman transport is driven by the horizontal velocity of the eddy, and operates along density fronts located on the eddy periphery, which is consistent across all eddy types [15, 17] (Fig 1). Nonlinear Ekman transport is also termed submesoscale transport, because it occurs at scales ranging from 0.1 to 10 km. In effect both Ekman and submesoscale vertical transport processes bring nutrients from mid-depths up to sunlit surface waters [23], stimulating primary production. Ekman upwelling within anticyclonic mode-water eddies has been used as a mechanism to explain massive phytoplankton blooms reported in the North Atlantic and elsewhere [24, 25]. Eddy-induced enhancement of chlorophyll concentrations at the eddy periphery as a result of submesoscale processes has also been observed [26–28]. Comparing the two vertical pumping mechanisms, submesoscale velocities operating on the eddy periphery can reach 10–100 m d^-1^, several orders of magnitude larger than velocities driven by Ekman transport occurring in the eddy center (0.1–0.4 cm d^-1^) [24, 29, 30]. Additionally, submesoscale processes can act to transport particulate organic carbon and oxygen downwards below the surface mixed layer, referred to as subduction [15, 20, 31–33] and this has recently been suggested to play a pivotal role in the ocean carbon pump [32]. Eddies are common throughout all major OMZs [34, 35], but the extent of their regulation over regional chlorophyll and the impact of this on nitrogen loss processes remains understudied.

To date, only a few studies have investigated the effect of eddies on nitrogen cycling processes in OMZs. These studies have used time-integrated records of nitrogen loss, such as natural abundance N-isotopes [14, 36], the nitrogen deficit (N*) [14, 37], and nitrite concentrations [36, 37]. All methods show signatures indicative of enhanced nitrogen loss and elevated chlorophyll, in the center of anticyclonic mode-water eddies, and this is referred to as the ‘hotspot’ theory [14, 36–38]. However, the current ‘hotspot’ theory is debated, because it assumes these chemical signatures originated and were intensified by the eddy, as a result of central Ekman upwelling [14, 36, 37]. This theory is contested, as it does not consider the eddy formation history and exchange with surrounding water bodies [39]. A study tracking the development of a coastal anticyclonic eddy in the ETSP region found that the eddy naturally entraps coastal water signatures, including coastally derived N* [39]. As the eddy continued developing, the signature was enhanced overtime by eddy-induced horizontal advective transport, in effect pulling coastally derived N* inwards towards its center [39]. Given that the coastal N* is typically higher than offshore waters [13], as the eddy propagated away from the coast it retained an elevated coastal signature offshore [20, 39]. Thomsen et al., [39] highlight that the accumulated biogeochemical signal preserves a record of water mass history, but does not necessarily indicate the presence of ongoing nitrogen loss activity. Likewise, the chlorophyll hotspot observed in anticyclonic mode-water eddies in the ETSP region and elsewhere, conventionally attributed to stimulation induced by central Ekman upwelling [24], could alternatively have accumulated in the eddy center as a result of inward horizontal transport [15, 40]. In contrast, recent studies outside of OMZs highlight that the most prevalent nutrient replenishment, and thereby stimulant of primary productivity, is occurring on the eddy periphery due to submesoscale dynamics [15, 26, 40–42].

We investigated the spatial distribution of nitrogen loss rates and chlorophyll across mesoscale eddies in the ETSP region. Specifically, we attempt to better resolve which vertical transport mechanisms regulate nitrogen loss and chlorophyll concentrations within eddies. In this study we provide the first *in situ* rate measurements of nitrogen loss across an eddy. Our analysis further expands our understanding of system wide patterns of offshore chlorophyll and the regulation of nitrogen loss as a result of the widespread distribution of eddies in the ETSP region.

## Results and Discussion

### Eddy hydrodynamics and station definition

**Fig 2:**
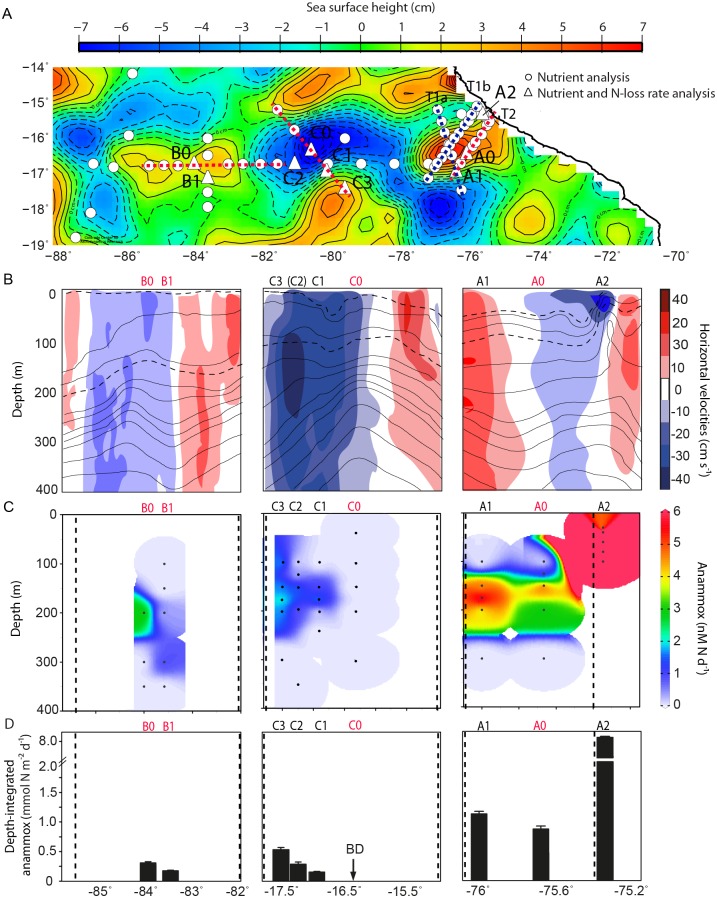
Distribution of anammox activity across eddies A, B and C in the ETSP region. (A) Sea surface height altimetry (SSHA) of sampled eddies A, B and C during the M90 cruise, November 22^nd^, 2012. The approximate locations of sampled stations within the eddy are shown, please note that the eddies propagated westward over the sampling period. Stations sampled for nutrients only (open circles) and nutrients plus nitrogen loss rates (open triangles with station numbers) are indicated. The red and blue dotted lines indicate transects sampled across eddies A, B and C, note that three defined transects were performed across eddy A (T1a/b in blue and T2 in red). Transects shown in panels B-D represent red dotted lines, whereas additional transects T1a (blue) are shown in S2–S3 Figs. (B) Horizontal velocity depth profiles are adapted from Stramma et al., [37]. Isopycnal contours are indicated by black lines, while reference isopycnals 25.4 and 26.0 kg m^-3^ are highlighted by black dotted lines. (C, D) Indicate volumetric and depth-integrated anammox rates for ^15^N-NH_4_^+^ experiments. Error bars for depth-integrated anammox rates represent the standard error. BD indicates below the limit of detection. Stations numbered in red (B0, B1, C0, and A0) were sampled in or near the eddy center while stations with black numbers (C3, C2, C1, and A1) were sampled on the eddy periphery, identified according to eddy-induced horizontal velocities, density fronts, and SSHA, shown in panels a and b. Note that the center of eddy C, based on SSHA and supported by horizontal velocities, is at -16.25°N, -80.38°E 14 km northeast of station C0. Note that data from station C2 is not included in the transect profiles shown in panel B (indicated by (C2)), but is shown in panels C, D. The coastal upwelling station is indicated by ‘A2’. The vertical black dotted lines in panels C and D indicate the edge of the respective eddies.

During the M90 research cruise in November 2012, eddies A, B and C, were readily observable from satellite sea surface height altimetry (SSHA), and were sampled along the 16.45°S transect (Fig 2A). Eddies A, B and C extended vertically from surface waters down to between 600–950 m depth [37]. At the time of sampling, eddy A was still forming over the upper shelf, whereas eddies B and C were detached from the coast and had propagated westward. Based on satellite altimetry tracking, eddy A was the youngest, followed by eddies C and B at 2, 3 and 5 months old, respectively [37]. Eddies A and B, based on isopycnal profiles were by definition anticyclonic mode-water eddies because they had uplifted seasonal pycnoclines and depressed main pycnoclines, whereas eddy C was cyclonic because it had upward shoaling of both seasonal and main pycnoclines ([24]; Figs 1 and 2B).

We define the location of our stations within the eddy according to two characteristic features: horizontal velocities for transects across individual eddies, and isopycnal spacing for system wide trends. 1) Horizontal velocities induced by the eddy vary across its diameter. Inherently, the center of the eddy has low or near-stagnant horizontal velocities that increase moving away in either direction from the center (Fig 2B). Horizontal velocities eventually peak and then decrease at the outer limits of the eddy. Generally, the center exhibits flat isopycnals that begin to tilt moving away in either direction from the center. The tilting of the isopycnals, otherwise referred to as the density front, coincides with an increase in eddy horizontal velocity (Fig 1). Unless specified we use the terms, “center” to describe the area of the eddy having the lowest horizontal velocity, and “periphery” referring broadly to the density front, which also coincides with higher eddy horizontal velocities. 2) For the analysis of system-wide trends, and specifically when horizontal velocity data was unavailable, stations were instead located according to the isopycnal spacing, following a similar concept as Strass [42]. In principle, on either side of each density front, isopycnals begin to flatten; notably, the center characteristically exhibits a smaller distance between individual isopycnals than the eddy periphery (Fig 1). Thereby, isopycnal spacing can be used to determine the relative location of sampled stations within an eddy (i.e. periphery versus center). Here, we use reference isopycnals, 25.4 and 26.0 kg m^-3^, to calculate isopycnal spacing. Isopycnals 25.4 and 26.0 kg m^-3^ located near the surface and oxycline, respectively, were chosen because they were representative of uplifted seasonal pycnoclines in eddies A, B and C (Fig 2B).

### Distribution of chemical parameters

Eddies A, B and C penetrated vertically through the OMZ core, which was observed between 100 and 500 m depths, using a cutoff of 20 μM oxygen (S1 Fig). These eddies had a distinct effect on the distribution of oxygen and nutrients (originally discussed by Stramma et al., [37]). For cyclonic eddy C, the center, had undetectable nitrite concentrations and an N* of -17 μM (S2 Fig). Chlorophyll concentrations were between 2–2.5 μg L^-1^ from 20 to 40 m depth, and oxygen concentrations were notably high, reaching more than 5 μM in the center from 200–350 m depth (S2–S4 Figs). At the periphery, chlorophyll concentrations were slightly lower (0.5–2 μg L^-1^) relative to the center, however, chlorophyll had a deeper penetration (down to 150 m) through the water column along the density front (S3 Fig). Increases in nitrite, N*, and a decrease in oxygen were observed moving away from the center along the density front. Furthermore, elevated concentrations and low oxygen waters (<3 μM) were observed over a larger depth range relative to the center. At the periphery, N* and nitrite concentrations were most pronounced, with values reaching -40 μM and 11 μM, respectively. Here it is important to note that these parameters (N* and nitrite concentrations) are traditionally thought of as chemical signatures of active nitrogen loss, but it has been shown that no quantitative correlation exists between them and ongoing nitrogen loss activity [8, 13, 43]. For eddy C, Stramma et al., [37] attributed the increase in nitrite and N* occurring along the periphery to an impinging anticyclonic eddy (seen in Fig 2A). An alternative interpretation is that strong upward directed transport of nutrients along the density front stimulated primary productivity, in agreement with modeling studies [40, 41]. The enhanced organic matter supply and subsequent remineralization decreased oxygen concentrations, and could potentially promote nitrogen loss activity.

For anticyclonic mode-water eddies A and B, nutrient and oxygen distributions across the eddy differed relative to eddy C. The center of eddy A had oxygen concentrations less than 3 μM between 140 and 400 m depth and a maximum chlorophyll concentration of 6.1 μg L^-1^ at 50 m depth (S2 and S3 Figs; Stramma et al., [37]). For eddy B, the maximum chlorophyll concentration was half that (2.5 μg L^-1^) of eddy A (S3 Fig), and oxygen-depleted (<3 μM) waters at the center were observed between 200 and 400 m depth (S1 and S2 Figs; [37]). N* and nitrite concentrations were most pronounced in the centers of eddies A and B, a strong contrast to eddy C (S2 Fig; [37]). Eddies A and B, exhibited a strong N* between 175–250 m depth of -30 μM, and nitrite concentrations up to 8 μM (S2 Fig). Moving away from the center of eddies A and B there was a decrease in nitrite and N*concentrations, as well as an increase in oxygen concentrations, indicating an opposite cross-eddy pattern between sampled cyclonic and anticyclonic mode-water eddies. Maximum chlorophyll concentrations also decreased moving towards the periphery, however, chlorophyll was distributed over a larger depth range, with lateral intrusions and/or deep penetrating pockets of chlorophyll being observed along the density front (S3 Fig; [37]). Similar features, occurring along the eddy periphery, have been observed in anticyclonic eddies in the North Atlantic, which were indicative of eddy-induced peripheral submesoscale transport processes [32]. In the study by Omand et al., [32] submesoscale vertical transport resulted in over half of the springtime bloom being exported below the surface mixed layer. Subducted chlorophyll in eddies A and B along the periphery could be considered evidence of active submesoscale driven transport, which may directly supply organic matter for nitrogen loss processes in the OMZ. Moreover in the ETSP region and elsewhere, submesoscale processes have been shown to introduce oxygen below the surface mixed layer [32, 33]. Consequently this could potentially fuel microaerobic activity that has been shown to be an important process in supplying ammonium for anammox bacteria [44].

### Distribution of nitrogen loss rates

To determine anammox and denitrification activity across each eddy we performed incubation experiments with ^15^N-NH_4_^+^ and ^15^N-NO_2_^-^ additions. Denitrification was below detection at all of the stations, which is in line with previous studies in the ETSP region, which have shown denitrification rates to be highly patchy [45]. Anammox activity dominated at the sampled stations, which is consistent with previous studies, suggesting anammox as the main microbial nitrogen loss pathway in the ETSP region [9, 13]. Volumetric anammox rates from the two incubation experiments were generally comparable to each other (S1 Fig). Over depth, the highest volumetric anammox rates generally corresponded with both N* and nitrite maximums, which also corresponded to the depths just below where oxygen dropped below 20 μM. Our volumetric anammox rates for two offshore stations and eddies A, B and C ranged from below detection to 8 nM N d^-1^, and for the coastal station ranged from below detection to 57 nM N d^-1^ (S1 Fig). These are comparable to previously reported anammox rates for coastal and offshore OMZ environments [9–13, 45]. Moreover, our volumetric rates followed the same longitudinal trend as Kalvelage et al., [13] indicating highest anammox activity over the shelf followed by a decrease of an order of magnitude at offshore stations. Although these volumetric rates are lower in the offshore OMZ, they exhibit large variability that is not related to the distance from the shelf [13].

To compare anammox activity across each eddy we will focus on the maximal volumetric and depth-integrated rates observed at each station, based on ^15^N-NH_4_^+^ incubations. For cyclonic eddy C, rates of anammox activity varied across the eddy transect, in total four stations were available for comparison. The center station (C0), which had the lowest horizontal velocity of the four stations, had non-detectable anammox activity (Fig 2C). The remaining three stations were sampled along the density front. The second (C1) and third (C2) closest stations to the center, located nearest to the highest horizontal velocity had anammox rates up to 1.55 and 1.71 nM N d^-1^ at 150 m depth. At the station furthest from the center (C3), activity increased to 2.11 nM N d^-1^, and had consistently high rates of 1.75 to 2.11 nM N d^-1^ between 100 and 200 m depth; this station corresponded to the outer edge of the density front where horizontal velocities began to decrease. The same trend is further highlighted when looking at the depth-integrated anammox rates, where we observed a transition from lowest to highest anammox rates moving from the center towards the periphery of the eddy, 0.00 to 0.53 ± 0.04 mmol N m^-2^ d^-1^ (Fig 2D). These findings indicate a tendency for anammox activity to increase moving away from the center across the eddy density front, and towards higher horizontal velocities.

For anticyclonic mode-water near-coastal eddy A volumetric anammox rates increased from 4.95 ± 0.50 nM N d^-1^ to 5.97 ± 0.50 nM N d^-1^, moving from the eddy center towards the periphery (Fig 2C), suggesting no across-eddy differences. However, depth-integrated anammox rates show a pattern identical to that of eddy C with lower activity in the eddy center (0.86 ± 0.05 mmol N m^-2^ d^-1^) relative to the periphery (1.12 ± 0.04 mmol N m^-2^ d^-1^; Fig 2D). Elevated anammox activity at the periphery coincided with the strongest horizontal velocities (Fig 2B). Anticyclonic mode-water eddy B, the weakest of the eddies based on its horizontal velocity, had nitrogen loss rates up to 3.04 nM N d^-1^, at two stations in close proximity to the eddy center (Fig 2B and 2C). With only center stations available it was not possible to determine if anammox rates were higher at the eddy periphery as observed for eddies A and C. Though, depth-integrated anammox activity in the center of eddy B was similar to that of an offshore station sampled at the same longitude (0.30 ± 0.02 and 0.39 ± 0.05 mmol N m^-2^ d^-1^ respectively, Fig 2D). This is notable, because the center of eddy B has previously been suggested as a ‘hotspot’ for nitrogen loss due to its elevated concentrations of N* and nitrite [37], but the direct measurements of ongoing activity measured here seem to disagree with this, as anammox rates were not higher in the center of eddy B.

The centers of cyclonic and anticyclonic mode-water eddies exhibited differences not only in nitrogen loss activity but also in nutrients. For cyclonic eddy C, anammox activity, nitrite, N*, and chlorophyll were lower at the center compared to the centers of eddies A and B. This difference in activity and nutrient distributions between eddies could potentially be explained by the direction of Ekman driven vertical transport at the eddy center. Anticyclonic mode-water eddies which produce upwelling would be expected to generate higher primary production and thereby higher organic matter export in the center than cyclonic eddies which drive nutrient downwelling [24]. Enhanced organic matter export could have fueled higher anammox activity in the centers of anticyclonic mode-water eddies A and B compared to cyclonic eddy C. Moreover, in eddy C, downwelling of oxygenated waters may have ventilated the eddy center, which could explain why oxygen concentrations never fell below 5 μM, while in eddies A and B concentrations were generally below the detection limit (3 μM). Recent studies quantifying the oxygen sensitivity of anammox have found 50% inhibition concentrations of 1 to ~10 μM oxygen [46, 47], which could be a potential explanation of why lower anammox activity was observed in the center of eddy C. Thus we suggest that the direction of Ekman driven vertical transport in the center plays a role in regulating nitrogen loss by controlling export production and oxygen supply.

While rates of nitrogen loss in the centers of eddies A and B were moderate, they were lower or comparable to rates observed at the periphery or other offshore stations, despite having elevated chlorophyll, N* and nitrite concentrations [37]. To date these enhanced chemical signatures of nitrogen loss (N*, nitrite concentrations and natural abundance N-isotopes) have been the basis for the hotspot theory, proposing Ekman upwelling as the main driver of nitrogen loss at the eddy center [14, 36, 37, 48]. However, as a consequence of how eddies form, mixing with adjacent waters could explain the majority of the nutrient and low oxygen concentrations. Indeed, salinity characteristics in the centers of eddies A and B were of a similar range to values measured for the coastal Peruvian-Chilean undercurrent (PCUC), ranging between 34.8–35.9 from 50 to 200 m water depth [37]. The coastal PCUC waters can vary in terms of nutrient chemistry, but generally have concentrations of nitrite, N*, and chlorophyll ranging between 6–9 μM, -25-28 μM, >6 μg L^-1^, respectively [13, 37, 39], as well as oxygen concentrations below 10 nM [3]. Conserved nutrient chemistry and salinity characteristics between the centers of eddies A and B and the PCUC would suggest that nutrients in the centers of eddies A and B originated from the coast. A similar finding was reported for another anticyclonic eddy occurring in the same region tracked over its formation history [39]. Thomsen et al [39], showed snapshots of nutrient concentrations before, during and after the eddy formation to reveal increasing nitrite and N* concentrations in the eddy center and decreasing oxygen over this period. After formation the eddy center had comparable nutrient concentrations to the PCUC. Moreover nutrient gradients (nitrate, nitrite and oxygen) formed along isopycnals between the eddy and the coast, diagnostic of eddy-induced horizontal advection [39]. In the ETSP region eddy-induced horizontal advection of coastal nutrients and productivity offshore lowers the overall productivity of the coastal upwelling region [49].

Contrary to the hotspot theory, our findings show that nitrogen loss activity at the periphery of eddies A and C is greater than activity at the eddy center (Fig 2). For eddy C, the increase in anammox activity along the periphery was also paralleled by increases in nitrite, N*, and chlorophyll as well as a decrease in oxygen concentrations [37]. Moreover, N_2_O, an intermediate of the denitrification pathway accumulated on the periphery of eddies A, B and C [38]. In high-resolution eddy models the periphery is the site of enhanced vertical nutrient replenishment, which by far exceeds vertical transport velocities induced by Ekman upwelling in the eddy center [15, 40]. The horizontal velocity of the eddy drives submesoscale transport that is predicted to occur along either side of the density front [15, 17]. Given that nitrogen loss is correlated with organic matter export [13], our nitrogen loss rates support the idea that for eddies A, and C the periphery is an important site supporting primary productivity and a supply of organic matter, which as a whole is driven by submesoscale transport, a previously unrecognized process regulating nitrogen loss.

### Large-scale trends: correlation of chlorophyll with eddy isopycnal spacing

**Fig 3:**
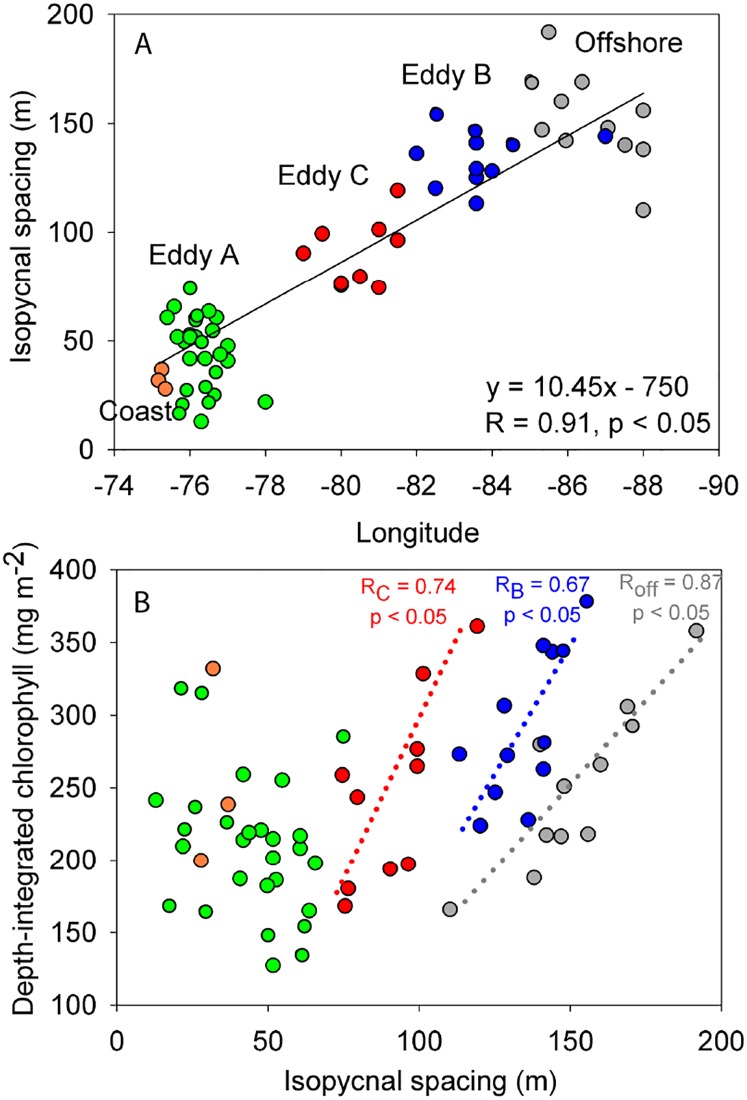
Relationship between isopycnal spacing and chlorophyll. (A) Correlation of isopycnal spacing versus longitude for eddies A (green), B (blue), and C (red), alongside coastal upwelling stations (orange) and offshore stations extending past eddy B (grey). (B) Correlation of isopycnal spacing versus depth-integrated chlorophyll. Chlorophyll at all stations was depth-integrated down to 300 m depth, except for coastal stations which were depth-integrated down to 200 m. Dotted linear regression lines indicate eddy specific trends (R_C_ = eddy C, R_B_ = eddy B and R_off_ = offshore). Pearson correlation values are indicated in each panel (p-values).

Isopycnal spacing, as previously mentioned, can be used to determine the relative position within an eddy (Fig 1). In general, isopycnal spacing is smallest at the eddy center and increases moving away in either direction along the density front (i.e. towards the eddy periphery; Fig 1). Thereby, we can use the relationship between isopycnal spacing and chlorophyll to identify patterns across an eddy. Additionally, isopycnal spacing conveys the approximate distance from the coast (plotted verses longitude in Fig 3A). Stations related to eddies A, B, and C group successively along this trend line with coastal and offshore stations found at either longitudinal extreme (R = 0.91, p < 0.05). This relationship with isopycnal spacing therefore provides an approximate location of the eddy across the longitudinal transect and the position within an eddy i.e. center vs. periphery.

In high-resolution chlorophyll profiles, mesopelagic intrusions and deep pockets can be seen extending into the surface mixed layer of all eddies, often occurring along the density front [37] (S3 Fig). Therefore, chlorophyll was depth-integrated at each station because of its broad vertical distribution. For offshore eddies C and B; depth-integrated chlorophyll was positively correlated with isopycnal spacing (Fig 3B; eddy C, R = 0.74, p < 0.05; eddy B, R = 0.67, p < 0.05). Interestingly, if we include in our analysis a range of offshore stations sampled along undefined transects past eddy B (Fig 3: grey circles), we find offshore stations produce a similar pattern to eddy C and B, signifying higher overall chlorophyll content with increasing isopycnal spacing (R = 0.87, p < 0.05). A different pattern emerged for the coastal anticyclonic mode-water eddy A, where no relationship was found between depth-integrated chlorophyll and isopycnal spacing. In other words no distinct pattern was observed in eddy A as stations grouped tightly together indicating that chlorophyll was evenly distributed across both the periphery and center of the mesoscale eddy (Fig 3B). Given the proximity of eddy A to the coast and our current understanding of horizontal advection induced by eddies [39], the lack of discernible difference in chlorophyll across eddy A could be ascribed to a masking effect caused by coastal-derived chlorophyll.

Plotting depth-integrated chlorophyll as a function of distance from the center of eddies A, B and C, based on SSHA also reveals depth-integrated chlorophyll to increase at the eddy periphery (S4 Fig; R = 0.50, p < 0.05). Notably, however, SSHA is not necessarily congruent with the subsurface properties of the eddy including the eddy horizontal velocity or isopycnal spacing [37, 39]. Arguably the more robust and less subjective method is to analyze depth-integrated chlorophyll as a function of isopycnal spacing. The finding of enhanced chlorophyll along the density front in this study (Fig 3 and S4 Fig), is also in agreement with high-resolution modeling studies, which demonstrate that submesoscale dynamics operate non-uniformly along the eddy density front creating pockets of upwelling and subduction [15, 17, 30, 40, 41]. An observational study by Strass, [42] has shown in a 2000 km transect across the North Atlantic a tendency for higher chlorophyll along the eddy density front where isopycnal spacing was largest and conversely lower chlorophyll concentrations when spacing was smallest. Evidence in this study indicates that peripheral chlorophyll extends deeper into the OMZ than at the center, as demonstrated by the appearance of lateral intrusions and deep chlorophyll pockets observed in eddy transect profiles ([37]; S3 Fig). Submesoscale processes may likewise play an important role in actively supplying organic matter in the offshore OMZ [33].

In addition to the coastally derived chlorophyll background (e.g. eddy C versus eddy A) our data further suggests that submesoscale peripheral processes have the potential to generate new chlorophyll. If we use chlorophyll as a proxy for primary production, then enhanced organic matter at the periphery, exported as either sinking particles or by subduction, could fuel measured anammox activity (Fig 3 and S3 Fig). Unfortunately, there is insufficient data available to perform a similar comparison of isopycnal spacing with depth-integrated anammox rates. Nevertheless, the relationship of chlorophyll with isopycnal spacing established over a large number of offshore stations, including stations sampled along undefined transects past eddy B is intriguing (Fig 3B). Why this holds could be attributed to the ubiquity of mesoscale eddies and submesoscale fronts, which have been shown to cause enhanced vertical transport in ETSP waters [33]. The combination of these processes, and their influence over vertical transport, could strongly regulate the distribution of chlorophyll in the ETSP region and thereby microbial nitrogen loss processes.

**Fig 4:**
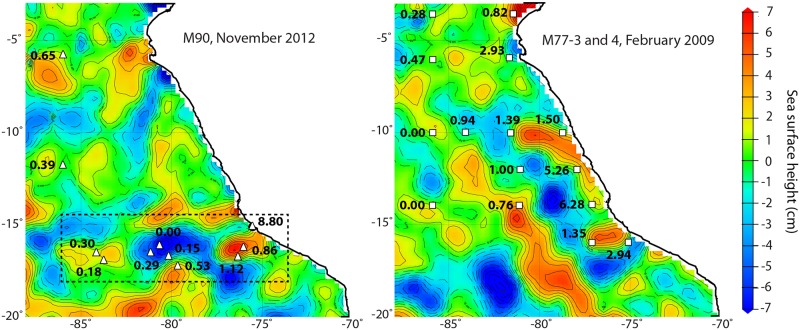
Widespread distribution of mesoscale eddies and the heterogeneity of anammox rates in the offshore ETSP region. Aerial sea surface height during the M90 (November 22^nd^, 2012) and the M77-4 (February 5^th^, 2009) research cruises. Eddies A, B and C shown in Fig 2A are highlighted by the dashed box in the left panel. Overlaid are depth integrated anammox rates (mmol N m^-2^ d^-1^) from ^15^N-incubation experiments from this study (left panel), and rates from the M77-3 and M77-4 research expeditions (right panel) [13]. Anammox rates are depth integrated over the OMZ at a cutoff of 20 μM oxygen.

Aerial sea surface height analysis highlights the widespread distribution of mesoscale eddies in the ETSP region. If we overlay depth-integrated anammox rates over sea surface height for stations sampled across eddies A, B and C, we find that nitrogen loss is heterogeneous (Fig 4). Similar heterogeneity in both nitrogen loss rates and the distribution of eddies was observed in previous ETSP sampling campaigns in January and February 2009 (Fig 4; M77-3 and -4; [13]), suggesting that eddies may drive much of the vertical nutrient transport and thereby primary productivity in the offshore OMZ. Previous studies in the ETSP region and elsewhere have shown that submesoscale transport is an important process, not only fueling enhanced primary productivity [40, 41], but also contributing to the subduction of organic matter below the surface mixed layer [32, 33]. Based on our findings we suggest that eddy-driven submesoscale vertical transport of nutrients and organic matter may be a major regulator of offshore ETSP nitrogen loss, which by volume represents the largest regional sink of fixed nitrogen.

## Summary and Conclusions

In this study we provide the first rate measurements of nitrogen loss processes across cyclonic and anticyclonic mode-water eddies in the ETSP. Contrary to the recent ‘hotspot’ studies, which have suggested that the highest activity occurs in the eddy center [14, 36–38, 48], our ^15^N-labelling incubation experiments revealed that nitrogen loss activity was greatest at the periphery of mesoscale eddies. Although, highest chlorophyll concentrations were observed in the center [37], depth-integrated chlorophyll content was also highest at the eddy periphery. The observed lateral intrusions and deep chlorophyll pockets occurring along the eddy periphery [37], suggest that this area of the eddy was active in the generation and export of organic matter, in agreement with modeling studies [40, 41].

Our findings, which indicate enhanced anammox activity and chlorophyll along the eddy periphery, appear to be consistent with these features being regulated by a submesoscale nutrient transport mechanism. The periphery of the eddy, as defined here and elsewhere, represents the eddy density front where isopycnals tilt and the spacing between isopycnals increases relative to the center. Specifically, submesoscale processes operate on either side of the density front, where the highest horizontal velocities occur [15, 17]. In other regions, eddy-induced submesoscale processes have been shown to be significant drivers of vertical nutrient transport along the eddy periphery, thereby providing a supply of organic matter below the surface mixed layer [32, 41], which then has the potential to fuel microbial nitrogen loss activity in OMZs. Observations from two additional sampling campaigns in the ETSP OMZ demonstrate heterogeneity in both mesoscale eddy activity and nitrogen loss rates. Together this is suggestive that eddy-driven vertical transport of nutrients may regulate offshore nitrogen loss.

On a global scale mesoscale eddies contribute to an estimated vertical water column nutrient flux of 0.12 mol N m^-2^ yr^-1^ [23, 50]. This estimation roughly doubles if global biogeochemical model simulations resolve for submesocale processes within eddies [51]. Current regional biogeochemical models, which have limited spatial resolution, do not yet include small-scale submesoscale features [52]. Parameterization of vertical mixing processes may thus help to improve biogeochemical models and provide a more realistic assessment of the marine OMZ nitrogen budget.

## Materials and Methods

### Ethics statement

Permission for the sampling campaign was obtained from the Peruvian authorities.

### Nutrient and hydrography analysis

Sampling was undertaken on the M90 research expedition onboard the R/V Meteor from October 31^st^ to November 26^th^, 2012. Eddies A, B and C were sampled along the 16.45’S transect (Fig 2A). Onboard, eddies were first identified and tracked by real-time SSHA data obtained from AVISO satellite altimetry. Transects through these eddies were made according to SSHA data. Horizontal velocities of the eddy were measured by acoustic Doppler current profiling (ADCP). A 75 and 38 kHz ADCP systems measured velocities down to 700 and 1200 m depth, respectively, detailed by Stramma et al., [37]. In this study we define eddy boundaries according to ADCP profiles and not specifically by SSHA.

Complete details of methods used to measure and analyze eddy nutrient chemistry are described elsewhere [37]. Briefly, a Seabird CTD-rosette equipped with 10L Niskin bottles was used to sample waters at depth. Chlorophyll, temperature, salinity, and oxygen were recorded by CTD sensors on both up and down casts. The oxygen sensor was calibrated by Winkler titration [53], with a detection limit of approximately 3 μM. Chlorophyll was calibrated according to the company specifications, with sensitivity down to 0.025 μg L^-1^. No shipboard chlorophyll calibration was applied, because of this, Stramma et al., [37] note that absolute numbers may have uncertainties; nevertheless, gradient trends observed across the eddy are accurate. Nutrient samples were taken to measure nitrate, nitrite, and phosphate onboard by a QuAAtro auto-analyzer (Seal Analytical), with precisions of ± 0.1 μmol L^-1^, ± 0.1 μmol L^-1^, and ± 0.02 μmol L^-1^, respectively. The N*, commonly used as a general measure of nitrogen loss, estimates from a given water mass chemistry the deviation of inorganic nitrogen pools from Redfield stoichiometry, was calculated according to the following equation N* = (NO_3_^-^ + NO_2_^-^) − 16PO_4_^3-^ (originally defined by [54], later modified by [14, 37]).

### ^15^N incubation experiments

*In situ* ^15^N-labelling incubation experiments were performed according to Holtappels et al., [55]. In brief, waters were sampled directly from the Niskin bottle into 250 mL glass serum bottles. Bottles were overflowed 2–3 times their volume and sealed headspace free with a butyl rubber stopper, that had been stored under helium for 2 days prior to use, to avoid oxygen contamination. Once filled, glass serum bottles were stored at *in situ* temperature in the dark until all depths were sampled. Each serum bottle was purged for a total of 15 min with helium; ^15^N-labeled isotopes were added with a gas-tight syringe after 5 min of purging to allow mixing. The experiments included the following additions: exp1: ^15^N-NO_2_^-^ + ^14^N-NH_4_^+^, and exp2: ^15^N-NH_4_^+^ + ^14^N-NO_2_^-^. The concentration of added substrates was 5 μM. After degassing, exetainers (12 mL, Labco, UK) were filled off and capped headspace free. Caps were degassed with a vacuum, followed by purging with helium three times and then stored 2–3 days before use, to reduce oxygen contamination [56]. Samples were incubated in the dark at *in situ* temperature. Exetainers were terminated at 0, 6, 12, 24 and 48 hours with 100 μL HgCl_2_ after inserting a 2 mL helium headspace. Terminated samples were stored in the dark at ambient temperature cap side down until further processing.

Isotope products ^14^N^15^N and ^15^N^15^N were measured by a gas-chromatography isotope-ratio mass spectrometer (GC-IRMS; VG Optima, Manchester, UK). The rates of N_2_ production from ^15^N-NH_4_^+^ and ^15^N-NO_2_^-^ incubation experiments were determined from the slope of the linear regression as a function of time. Anammox and denitrification rates were calculated according to the equations of Thamdrup and Dalsgaard, [57]. A t-test was used to determine whether rates were significantly different from zero (p < 0.05). Detection limits were estimated from the median of the standard error of the slope, multiplied by the t-value for p = 0.05, thus the detection limits for anammox were 0.68 and 0.66 nM N d^-1^ for ^15^N-NH_4_^+^ and ^15^N-NO_2_^-^ incubation experiments, respectively. The majority of our analysis is based on the ^15^N-NH_4_^+^ incubations, due to the potential caveats of using ^15^N-NO_2_^-^ to determine anammox rates; nitrogen isotope exchange between the nitrate and nitrite pools [58], and ‘nitrite shunting’ [59].

Anammox rates were depth integrated from the base of the upper oxycline down to the bottom oxycline (using an oxygen cutoff of 20 μM), analogous to depth integrated rates reported by Kalvelage et al., [13]. At all offshore stations chlorophyll was depth-integrated down to 300 m depth, which was the deepest depth reported for anammox activity in eddies A and C. At coastal stations chlorophyll was depth-integrated down to 200 m. Isopycnal spacing was calculated for each station by subtracting the distance between reference densities 25.4 and 26.0 kg m^-3^. Pearson correlation statistics were applied to determine if relationships were significant (p < 0.05).

## Supporting Information

S1 Fig Depth profiles of anammox activity, nutrient, and oxygen concentrations at stations sampled within eddies A, B and C, and two offshore stations. The location of stations is indicated in Fig 2A and S1 Table. Anammox activity for ^15^N-NH_4_^+^ and ^15^N-NO_2_^-^ experiments are indicated in separate panels. For stations B0 and B1 (eddy B) anammox rates from ^15^N-NO_2_^-^ experiments were not determined. Error bars for anammox rates represent the standard error. The N-deficit was calculated according to Stramma et al., [37], see material and methods section.
(TIF)

S2 Fig Distribution of oxygen, N*, and nutrients across eddies A, B and C in the ETSP region. The cross eddy transects are shown in Fig 2A. Note that both oxygen, N*, and nutrients transects of eddy A are indicated (see Fig 2A for transect stations: T1a (blue dotted lines) and T2 (red dotted lines)). Stations numbered in red (B0, B1, C0, and A0) were sampled in the eddy center while stations with black numbers (C3, C2, C1, and A1) were sampled on the eddy periphery, identified according to eddy-induced horizontal velocities and density fronts, shown in Fig 2. Note that data from station C2 is not included in the transect profiles shown (indicated by (C2)). The coastal upwelling station is indicated by ‘A2’. The vertical black dotted lines in panels A-D represent the outer periphery of the respective eddies. Data shown is adapted from Stramma et al., [37].
(TIF)

S3 Fig Distribution of chlorophyll across eddies A, B and C in the ETSP region. The cross eddy transects are shown in Fig 2A. Note that both chlorophyll transects of eddy A are indicated (see Fig 2A for transect stations: T1a (blue dotted lines) and T2 (red dotted lines)). Stations numbered in red (B0, B1, C0, and A0) were sampled in the eddy center while stations with black numbers (C3, C2, C1, and A1) were sampled on the eddy periphery, identified according to eddy-induced horizontal velocities and density fronts, shown in Fig 2. Note that data from station C2 is not included in the transect profiles shown (indicated by (C2)). The coastal upwelling station is indicated by ‘A2’. The vertical black dotted lines in each panel represent the outer periphery of the respective eddies. Data shown is adapted from Stramma et al., [37].
(TIF)

S4 Fig Distribution of depth-integrated chlorophyll across eddies A, B and C in the ETSP region based on satellite sea surface height altimetry (SSHA). (A) Aerial SSHA snapshot of eddies A, B and C. The eddy center is marked by the red cross, determined based on SSHA and the stations indicated are the same stations as those used in Fig 3 (offshore stations are not included). Note that eddy A is subdivided into three distinct transects (T1a/b and T2), with transects 1 and 2 having a different eddy center (red cross) as the transects were sampled approximately 5 days apart, and the eddy had propagated westward during this time. (B) Depth-integrated chlorophyll plotted as a function of distance from the eddy center. Depth-integrated chlorophyll of stations located a similar distance from the center (±2 km) were averaged, as indicated by the error bars (the standard error is shown). The overlaid dotted lines indicate the average depth-integrated chlorophyll for the eddy center, periphery and outside the eddy. Chlorophyll at all stations was depth-integrated down to 300 m depth, except for coastal stations which were depth-integrated down to 200 m. Plotting depth-integrated chlorophyll in panel B as a function of distance from the eddy center for all eddy stations (excluding the outside eddy stations) indicates a significant positive correlation (R = 0.50, p < 0.05).
(TIF)

S1 Table List of stations sampled for anammox rates during the M90 research cruise November 2012. (PDF)
