# Supplementary figures and images for: Enhanced Nitrogen Loss by Eddy-Induced Vertical Transport in the Offshore Peruvian Oxygen Minimum Zone
Source: PLoS One. 2017 Jan 25;12(1):e0170059. doi: 10.1371/journal.pone.0170059 (PMC5266280; doi:10.1371/journal.pone.0170059)

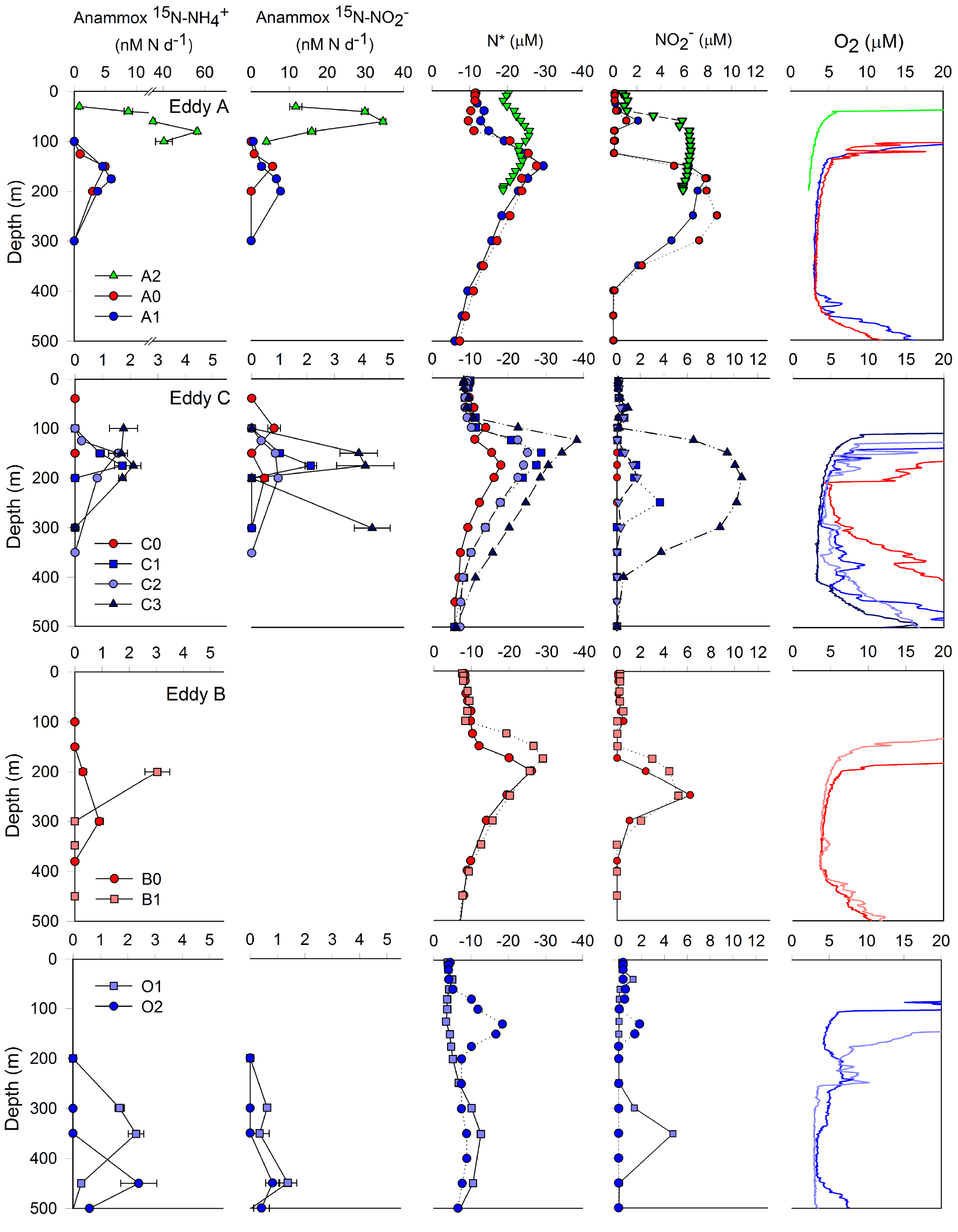

Supplement: S1 Fig — The location of stations is indicated in Fig 2A and S1 Table. Anammox activity for 15N-NH4+ and 15N-NO2- experiments are indicated in separate panels. For stations B0 and B1 (eddy B) anammox rates from 15N-NO2- experiments were not determined. Error bars for anammox rates represent the standard error. The N-deficit was calculated according to Stramma et al., [37], see material and methods section. (TIF) [file pone.0170059.s001.tif]

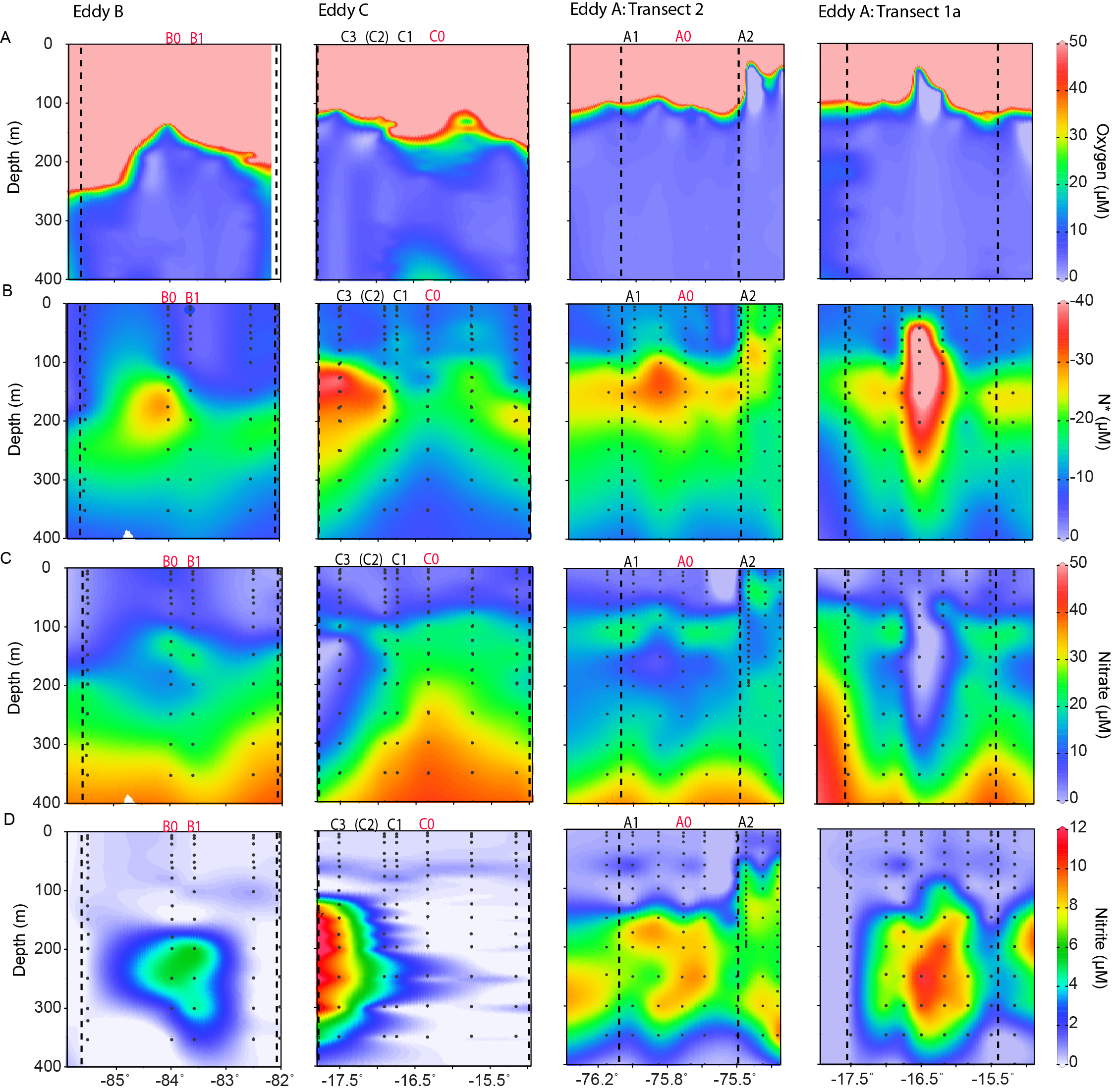

Supplement: S2 Fig — The cross eddy transects are shown in Fig 2A. Note that both oxygen, N*, and nutrients transects of eddy A are indicated (see Fig 2A for transect stations: T1a (blue dotted lines) and T2 (red dotted lines)). Stations numbered in red (B0, B1, C0, and A0) were sampled in the eddy center while stations with black numbers (C3, C2, C1, and A1) were sampled on the eddy periphery, identified according to eddy-induced horizontal velocities and density fronts, shown in Fig 2. Note that data from station C2 is not included in the transect profiles shown (indicated by (C2)). The coastal upwelling station is indicated by ‘A2’. The vertical black dotted lines in panels A-D represent the outer periphery of the respective eddies. Data shown is adapted from Stramma et al., [37]. (TIF) [file pone.0170059.s002.tif]

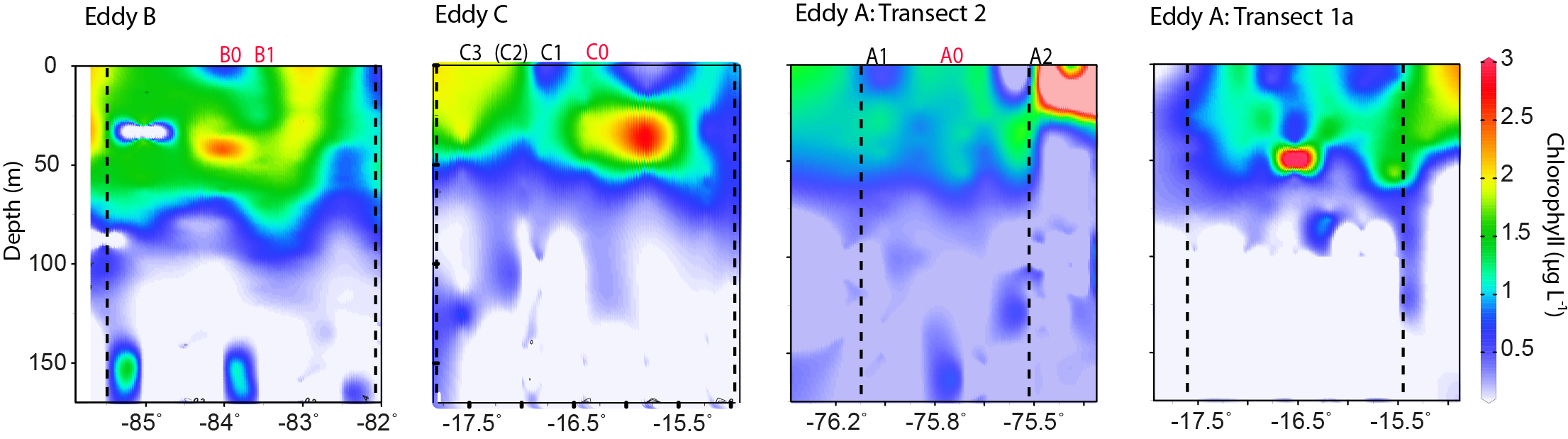

Supplement: S3 Fig — The cross eddy transects are shown in Fig 2A. Note that both chlorophyll transects of eddy A are indicated (see Fig 2A for transect stations: T1a (blue dotted lines) and T2 (red dotted lines)). Stations numbered in red (B0, B1, C0, and A0) were sampled in the eddy center while stations with black numbers (C3, C2, C1, and A1) were sampled on the eddy periphery, identified according to eddy-induced horizontal velocities and density fronts, shown in Fig 2. Note that data from station C2 is not included in the transect profiles shown (indicated by (C2)). The coastal upwelling station is indicated by ‘A2’. The vertical black dotted lines in each panel represent the outer periphery of the respective eddies. Data shown is adapted from Stramma et al., [37]. (TIF) [file pone.0170059.s003.tif]

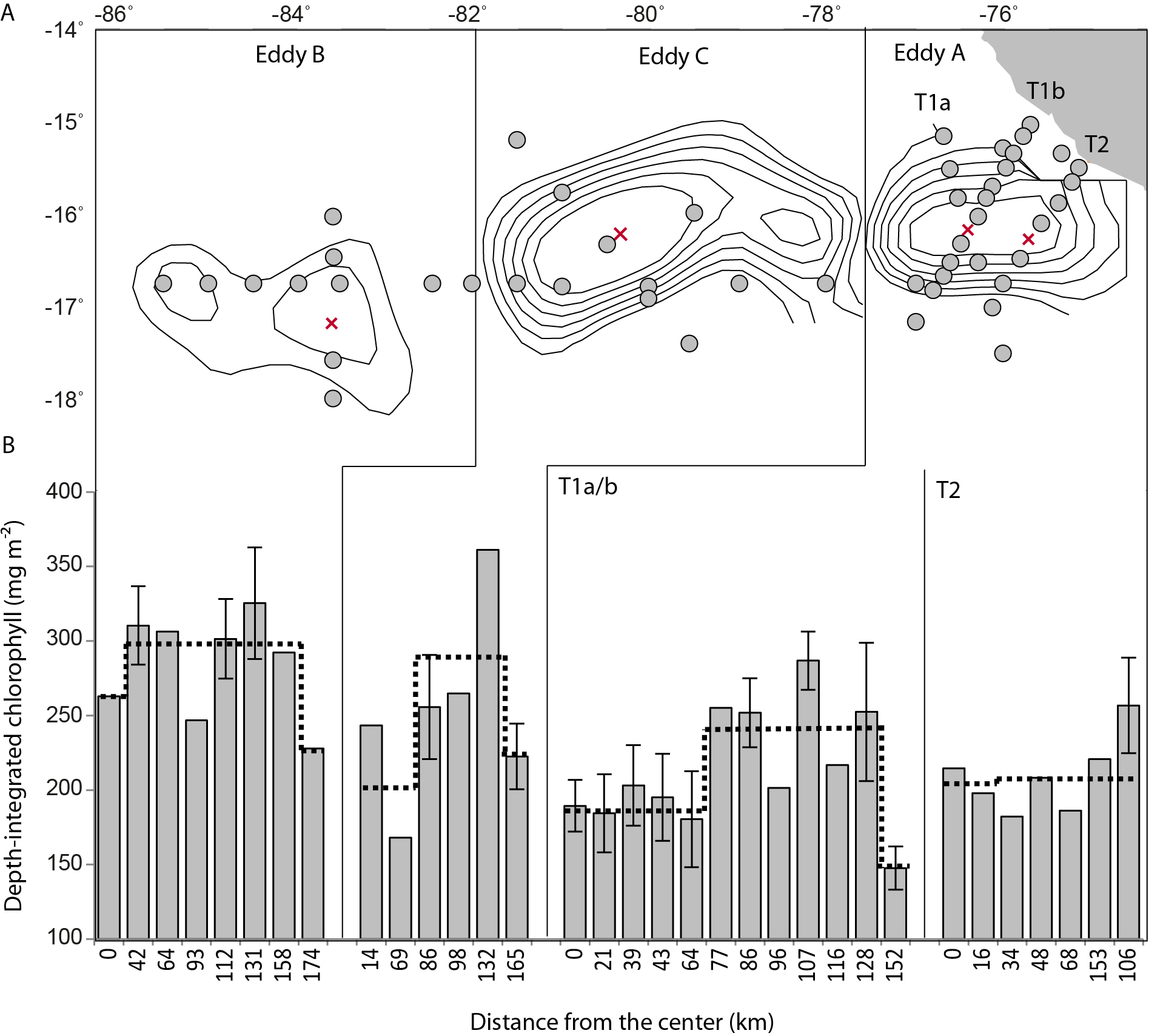

Supplement: S4 Fig — (A) Aerial SSHA snapshot of eddies A, B and C. The eddy center is marked by the red cross, determined based on SSHA and the stations indicated are the same stations as those used in Fig 3 (offshore stations are not included). Note that eddy A is subdivided into three distinct transects (T1a/b and T2), with transects 1 and 2 having a different eddy center (red cross) as the transects were sampled approximately 5 days apart, and the eddy had propagated westward during this time. (B) Depth-integrated chlorophyll plotted as a function of distance from the eddy center. Depth-integrated chlorophyll of stations located a similar distance from the center (±2 km) were averaged, as indicated by the error bars (the standard error is shown). The overlaid dotted lines indicate the average depth-integrated chlorophyll for the eddy center, periphery and outside the eddy. Chlorophyll at all stations was depth-integrated down to 300 m depth, except for coastal stations which were depth-integrated down to 200 m. Plotting depth-integrated chlorophyll in panel B as a function of distance from the eddy center for all eddy stations (excluding the outside eddy stations) indicates a significant positive correlation (R = 0.50, p < 0.05). (TIF) [file pone.0170059.s004.tif]
