# Supplementary material for: Enhanced Nitrogen Loss by Eddy-Induced Vertical Transport in the Offshore Peruvian Oxygen Minimum Zone
Source: PLoS One. 2017 Jan 25;12(1):e0170059. doi: 10.1371/journal.pone.0170059 (PMC5266280; doi:10.1371/journal.pone.0170059)
Supplement: S1 Table — (PDF) [file pone.0170059.s005.pdf]

Abbreviated station name (used in text)	M90 station name	Latitude (°N)	Longitude (°E)
B0	1639	-16.75	-84.00
B1	1646	-17.17	-83.58
C0	1659	-16.33	-80.50
C1	1660	-16.92	-80.00
C2	1652	-16.75	-81.00
C3	1661	-17.50	-79.50
A0	1672	-16.23	-75.67
A1	1668	-16.74	-76.00
A2	1679	-15.33	-75.35
O1	1581	-6.00	-85.83
O2	1604	-12.00	-85.83
